# Targeting the astrocytic metabolic cascade in Alzheimer’s disease: mechanisms, challenges and opportunities

**DOI:** 10.3389/fnagi.2026.1767811

**Published:** 2026-03-04

**Authors:** Huawen Cao, Junyi Liang, Xiaohong Dong, Zhiqi Xia, Xiaoting Luo, Bin Liu

**Affiliations:** 1 Heilongjiang University of Traditional Chinese Medicine, Harbin, Heilongjiang, China; 2 Heilongjiang University of Traditional Chinese Medicine, Jiamusi College, Jiamusi, Heilongjiang, China

**Keywords:** Alzheimer’s disease, amyloid-β metabolism, astrocytes, metabolic cascade, neuroinflammation, synaptic integrity, tau hyperphosphorylation

## Abstract

Alzheimer’s disease (AD), a pressing global public health challenge, is underpinned by multifaceted pathogenic mechanisms. While traditional research has centered on amyloid-β deposition and tau hyperphosphorylation, emerging evidence reveals that metabolic perturbations play a pivotal role in the earliest phases of AD. As the principal regulators of energy homeostasis within the central nervous system, astrocytes orchestrate a multistep metabolic cascade—encompassing glucose uptake, glycolysis, mitochondrial oxidative metabolism, and the release of metabolic intermediates—to sustain neuronal energy supply and synaptic integrity. In the AD milieu, this astrocytic metabolic cascade becomes profoundly disrupted at every level. Such metabolic dysregulation not only compromises the neuroprotective functions of astrocytes but also directly accelerates synaptic degeneration, exacerbates Aβ and tau pathologies, and amplifies neuroinflammatory responses, collectively forming a core “metabolic-neurodegeneration” pathological axis. Here, we provide a comprehensive synthesis of the aberrant astrocytic metabolic cascade in AD, delineating its critical contributions to synaptic deterioration, proteinopathy progression, and inflammatory escalation. Building on these insights, we propose a conceptual model of an “astrocyte-centric metabolic collapse,” highlighting metabolic derailment as a fundamental initiating and amplifying force in AD pathogenesis. Furthermore, we evaluate therapeutic strategies targeting key nodes of this cascade and discuss the challenges and opportunities inherent in modulating astrocytic metabolism. Through integrating the most recent advances, this review offers a refined understanding of astrocytic metabolic dysregulation in AD and examines its potential as a promising avenue for therapeutic intervention.

## 1 Introduction

Alzheimer’s disease (AD), the primary cause of dementia affecting more than 55 million people worldwide, imposes a substantial public health burden (Alzheimers Dement, 2024). Although Aβ deposition, tau hyperphosphorylation, and synaptic loss define its classical pathology, the cellular disturbances that initiate these events remain unclear. Converging evidence now positions metabolic dysfunction as an early and pivotal feature of AD pathogenesis (Yu et al., 2022; Zheng and Wang, 2025). The brain’s high energy demand—consuming ~20% of systemic glucose—renders it particularly sensitive to metabolic perturbation, and pronounced hypometabolism is detectable in prodromal AD, often preceding measurable Aβ accumulation (Bonvento and Bolanos, 2021; Yu et al., 2022; Wang et al., 2022). This early metabolic impairment is increasingly recognized not merely as a biomarker but as a mechanistic driver of disease progression.

Astrocytes - the most abundant glial population - serve essential functions in maintaining neuronal energy homeostasis, neurotransmitter turnover, and blood–brain barrier integrity (Brandebura et al., 2023). Under physiological conditions, astrocytes import glucose through GLUT1, metabolize it via glycolysis, and export lactate to neurons through the astrocyte–neuron lactate shuttle (ANLS) to support oxidative metabolism (Beard et al., 2021). In AD, however, this glucose-dependent metabolic cascade is markedly disrupted. Single-nucleus transcriptomic profiling of human AD brains reveals widespread dysregulation of astrocytic metabolic pathways, indicating an early breakdown of glial energy processing (Fan et al., 2023). Such metabolic impairment not only precedes classical histopathology but may also directly contribute to cognitive decline by destabilizing neuron–glia metabolic coupling (Andersen et al., 2021c; Suzuki et al., 2011).

In this Review, we examine the astrocytic glucose metabolic cascade as both a vulnerable target and an active driver of AD pathogenesis. We synthesize current evidence on how astrocytic metabolic dysfunction shapes neuronal vulnerability, accelerates neurodegeneration, and influences disease trajectory. We further highlight emerging therapeutic strategies aimed at restoring astrocyte-dependent metabolic support, thereby offering new conceptual and translational avenues for AD intervention.

## 2 Physiological roles of astrocytic glucose metabolism

### 2.1 Energy substrate management by astrocytes

The blood–brain barrier (BBB) forms a highly selective interface that governs molecular exchange between the circulation and neural tissue (Aborode et al., 2025). Astrocytic endfeet, integral components of the neurovascular unit, envelop the cerebral microvasculature and contribute to both barrier function and the regulation of cerebral blood flow (Preininger and Kaufer, 2022). Glucose transport into the brain is mediated predominantly by GLUT1, which is abundantly expressed on the luminal and abluminal surfaces of endothelial cells. Astrocytic endfeet also express high levels of GLUT1, establishing a coordinated glucose delivery route from the vasculature to astrocytes (Koepsell, 2020; Schiera et al., 2024).

Astrocytes serve as the principal reservoir of glycogen in the adult brain (Beltran-Velasco, 2025). Mobilization of this glycogen provides a rapid energy buffer during periods of heightened neuronal demand and supplies lactate through the ANLS to sustain synaptic transmission (Chen Z. et al., 2023). This capacity positions astrocytes as key regulators of energy substrate availability under both physiological and metabolically stressful conditions.

### 2.2 Astrocytic networks of metabolic coupling

Metabolic coupling between astrocytes and neurons is essential for maintaining cerebral energy homeostasis, with the ANLS representing its core mechanism (Kim et al., 2025). Astrocytes express MCT1 and MCT4 together with LDHA to export glycolytically derived lactate, whereas neurons rely on MCT2 and LDHB to import and oxidize lactate for ATP generation (Coca et al., 2025; Lee J. S. et al., 2025; Kim et al., 2025). Through this division of labor, astrocytes function as metabolic gatekeepers that match neuronal energy supply to activity demands. Nevertheless, the functional significance of ANLS remains a subject of debate. Measurements of cerebral oxygen consumption using BOLD fMRI and related approaches have suggested that glucose remains the indispensable substrate for brain energy metabolism, thereby questioning the efficiency of lactate-based energy transfer and the quantitative contribution of lactate transport and oxidation under physiological conditions (Schurr, 2025).

Beyond metabolic support, astrocytes exert broad regulatory control over neural circuit development and function. They secrete glia-derived neurotrophic factors and lipid precursors such as cholesterol, which are essential for oligodendrocyte precursor differentiation and myelination, and form perisynaptic astrocytic processes that directly participate in synapse formation, maturation and elimination across the synaptic life cycle (Chung et al., 2024; Tan et al., 2023). The tripartite synapse exemplifies this integrative neuron–astrocyte signaling unit. Glutamate released during synaptic activity activates NMDA and AMPA receptors to drive Ca^2+^ influx and long-term potentiation (LTP), thereby encoding learning and memory (Basavaraju and Priyadarshini, 2025). However, excessive extracellular glutamate induces excitotoxicity and neuronal injury (Lee et al., 2022). Astrocytes prevent this by rapidly clearing synaptic glutamate via EAAT1/2 and converting it to glutamine through glutamine synthetase, thus completing the glutamate–glutamine cycle (Andersen and Schousboe, 2023a; Todd and Hardingham, 2020).

Importantly, glutamate uptake is tightly coupled to astrocytic glucose metabolism (Pellerin and Magistretti, 1994). The Na^+^ influx accompanying EAAT-mediated transport activates Na^+^/K^+^-ATPase, increasing ADP/AMP levels and subsequently stimulating glycolysis, partly via AMPK-dependent pathways (Guillem et al., 2022; Muraleedharan and Dasgupta, 2022; Steinberg and Hardie, 2023). This metabolic activation enhances lactate production and directly fuels the ANLS, forming a coordinated “glutamate uptake–glycolysis–lactate release” mechanism that links neurotransmission to neuronal energy provision. In parallel, astrocytes channel glucose into the pentose phosphate pathway to maintain glutathione (GSH) pools, thereby supporting neuronal antioxidant defense.

## 3 Disruption of the glucose metabolic cascade in AD

**Figure 1:**
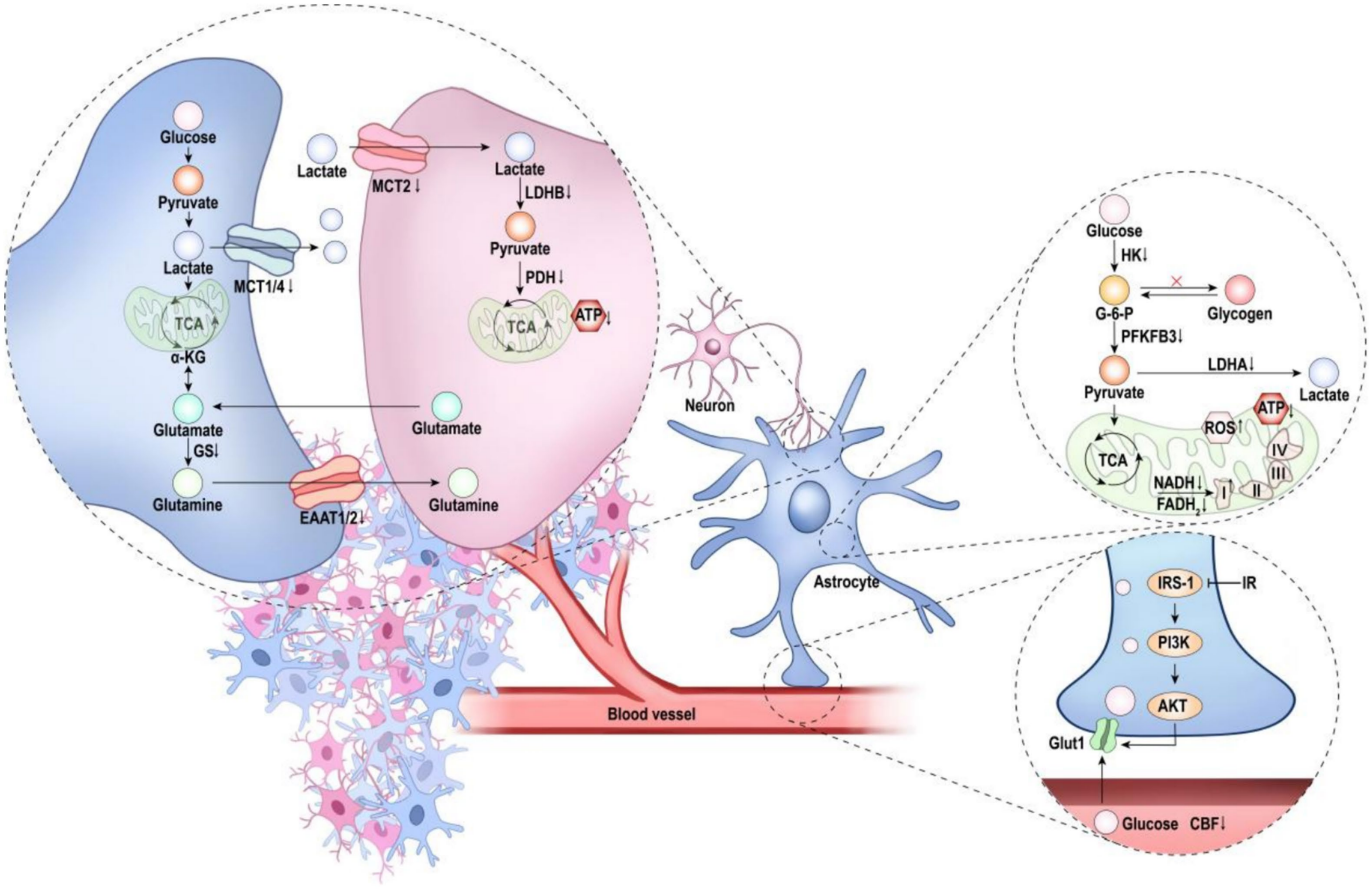
Dysregulated astrocytic glucose-metabolic cascade in AD. Astrocytes exhibit a global metabolic derailment encompassing metabolic input, intracellular pathways, and metabolic output. Pathological alterations within the neurovascular unit reduce cerebral blood flow and impair glucose transport; diminished GLUT1 expression further restricts glucose entry into astrocytes. Within metabolic pathways, glycolytic enzymes are downregulated, and mitochondrial dysfunction leads to reduced lactate production, diminished ATP generation, and excessive ROS accumulation. At the output level, impaired lactate transport decreases lactate availability to neurons, ultimately precipitating neuronal energy deficits. In addition, the downregulation of EAAT1/2 disrupts the glutamate–glutamine cycle, further compromising neuronal homeostasis.

Transcriptomic and functional studies reveal that astrocytes undergo pronounced phenotypic remodeling during ageing, with these alterations further exacerbated in AD (Fan et al., 2023). Although astrocytes clearly participate in AD pathogenesis, whether their actions are protective, deleterious, or context-dependent remains unresolved. A growing body of evidence indicates that the astrocytic glucose metabolic cascade becomes progressively compromised in AD (Figure 1; Table 1). Given the central role of this cascade in energy allocation, neurotransmitter cycling, and synaptic maintenance, its disruption constitutes a major driver of disease progression. Restoring astrocytic metabolic integrity therefore represents a compelling therapeutic strategy.

**Table 1:** Astrocyte glucose metabolism cascades: dysregulation mechanisms and manifestations in AD.

Dysregulation level	Mechanism	Manifestation	Refs.
Metabolic input collapse	GLUT1 dysfunction	↓GLUT1 expression on astrocytes; reduced cerebral glucose uptake	Bell et al. (2025), Hendrix et al. (2021)
	Insulin signaling impairment	↓IRS1/2, PI3K, Akt phosphorylation; impaired GLUT1 regulation	Bentivegna et al. (2025), Barthel et al. (1999)
	Cerebrovascular lesions	↓CBF; reduced glucose delivery to parenchyma	Thieren et al. (2025), Sheng et al. (2025)
Metabolic pathway disruption	Glycolysis downregulation	↓HK, LDHA protein levels; reduced glycolytic flux	Bell et al. (2025), Jia et al. (2025)
	Mitochondrial dysfunction	↑Mitochondrial fragmentation; ↓TCA activity; ↓NADH/FADH₂; ↑ROS	Andersen and Skotte et al. (2021c), Chen W. et al. (2023), Mohan and Kumar (2025), Pradeepkiran and Reddy (2020), Zysk et al. (2023)
	Glycogen storage deficit	↓Glycogen synthase activity; reduced glycogen	Bak et al. (2018)
Metabolic output failure	ANLS collapse	↓Lactate; ↓MCT1/2/4 expression; ↓LDHB, PDH activity	Jiang et al. (2023), Le Douce et al. (2020), Sakimura et al. (2024), Wang et al. (2024), Xu et al. (2016), Zhang et al. (2018)
	Glutamate–glutamine cycle dysregulation	↓TCA, ↓glutamine synthesis, ↓GS activity; ↓EAAT1/2; ↑extracellular Glu → excitotoxicity	Andersen et al. (2021b), Andersen et al. (2021c), Andersen et al. (2022), Barros et al. (2023), Wu et al. (2025)
Gut–brain axis regulation	SCFAs (butyrate, acetate, propionate)	BBB modulation; ↑MCT1/4, ↑GS; enhanced glutamine–glutamate shuttle	Jung et al. (2024), Silva et al. (2020), Zhang L. et al. (2025)
	TMAO/LPS	Astrocyte activation; pro-inflammatory phenotype; impaired BBB; insulin resistance	Abildinova et al. (2024), Peng et al. (2021), Yang et al. (2024), Su et al. (2021), Zhao et al. (2021)

### 3.1 Collapse of the metabolic input layer

As the principal metabolic intermediary between the BBB and neurons, astrocytes rely on efficient glucose uptake to sustain neuronal energy homeostasis. In AD, this capacity deteriorates early and profoundly.

Astrocytic glucose uptake is mediated largely by GLUT1, which is abundantly expressed at astrocytic endfeet and endothelial interfaces (Koepsell, 2020). Studies have demonstrated that astrocytes differentiated from induced neural progenitor cells (iNPCs) derived from patients with AD, as well as astrocytes from AD mouse models, exhibit marked reductions in GLUT1 protein abundance and aberrant membrane localization, directly impairing glucose uptake efficiency (Bell et al., 2025; Hendrix et al., 2021). Using an abdominal surgery model in aged wild-type mice, Chen Y. et al. (2023) further showed that GLUT1 downregulation disrupts cerebral glucose metabolism and robustly precipitates postoperative cognitive dysfunction, thereby establishing a causal link between impaired GLUT1 expression and cognitive decline. Notably, reductions in cerebral GLUT1 can be detected at early stages of AD, and its functional insufficiency accelerates Aβ pathology and disease progression (Winkler et al., 2015). However, despite these observations, the direct contribution of astrocyte-specific GLUT1 loss to AD pathogenesis remains insufficiently explored. Impaired cerebral insulin signalling represents a second major pathogenic axis. Widespread brain insulin resistance is a defining feature of AD (Kshirsagar et al., 2021), and astrocytes display a pronounced, cell-type-specific attenuation of insulin receptor (IR) activation together with reduced phosphorylation and signalling efficiency of downstream effectors, including PI3K and Akt (Bentivegna et al., 2025). This signalling deficit compromises insulin-dependent regulation of GLUT1 (Barthel et al., 1999) and further exacerbates defects in glycolysis and mitochondrial function, thereby amplifying metabolic vulnerability in the AD brain (Chen W. et al., 2023).

Cerebrovascular abnormalities—including amyloid angiopathy, chronically reduced cerebral blood flow, and impaired neurovascular coupling—further restrict glucose delivery (Custodia et al., 2023; Gao et al., 2025; Lourenco et al., 2017; Thal and Gawor, 2023). Because astrocytic endfeet closely monitor and respond to vascular substrate supply, vascular insufficiency synergizes with intrinsic transporter deficits to impair metabolic input (Thieren et al., 2025; Sheng et al., 2025). Approaches that restore cerebrovascular function, such as remote ischemic conditioning, can enhance GLUT1 expression and may offer metabolic benefits (Ma et al., 2024).

### 3.2 Disruption of glycolytic and mitochondrial metabolic pathways

After entering astrocytes, glucose is partitioned into cytosolic glycolysis and mitochondrial oxidative metabolism. In AD, both pathways exhibit profound and multifaceted disruption.

*Glycolytic dysfunction*: Glycolytic regulation in AD astrocytes displays a biphasic trajectory. Single-cell transcriptomic studies reveal early upregulation of glycolytic genes—possibly reflecting a compensatory response—followed by their downregulation at later stages (Serrano-Pozo et al., 2024). Excessive glycolysis during reactive astrocytosis may facilitate a detrimental phenotype (Bell and Mortiboys, 2026; Sanchez et al., 2025). Aβ-mediated inhibition of the key glycolytic regulator PFKFB3 impairs stress-responsive glycolytic upregulation, increasing astrocyte vulnerability and exacerbating plaque deposition (Almeida et al., 2023; Ahmad et al., 2023). Integrated analyses of AD brain transcriptomes further reveal downregulation of core glycolytic enzymes, including GPI, PFKM and LDHA, changes that correlate with cognitive decline (Jia et al., 2025). Consistently, astrocytes differentiated from sporadic and familial AD (sAD/fAD) patient-derived iNPCs display impaired glycolytic capacity accompanied by reduced HK1 expression (Bell et al., 2025). Notably, experimental enhancement of aerobic glycolysis in astrocytes has been shown to improve neuronal metabolic support, promoting neuronal survival and axonal outgrowth (Zheng et al., 2021). Nevertheless, both excessive and insufficient glycolytic activity appear detrimental, underscoring that dysregulated—rather than directionally altered—glycolysis is a driver of AD pathogenesis.

*Mitochondrial dysfunction*: Mitochondrial abnormalities constitute a persistent antecedent of AD pathology (Maruthiyodan et al., 2024). Astrocytes from patients with AD and from AD mouse models exhibit pronounced defects in mitochondrial dynamics, characterized by increased fragmentation and disrupted cristae architecture, culminating in progressive mitochondrial dysfunction (Pradeepkiran and Reddy, 2020; Zysk et al., 2023). The tricarboxylic acid (TCA) cycle, a central metabolic pathway within the mitochondrial matrix, is markedly suppressed in astrocytes from 5xFAD mice, with concomitant reductions in NADH and FADH₂ levels, thereby impairing downstream oxidative phosphorylation (OXPHOS) and enhancing oxidative stress (Andersen et al., 2021c; Mohan and Kumar, 2025). Mitophagy serves as a critical quality-control mechanism by selectively eliminating depolarized, damaged or aged mitochondria to prevent their accumulation (Wang et al., 2026). However, studies in 5xFAD mice demonstrate that astrocyte-specific insulin signalling deficiency reduces autophagic flux and perturbs mitophagy, thereby aggravating mitochondrial dysfunction and accelerating disease progression (Chen W. et al., 2023). In parallel, downregulation of astrocytic GLUT1 markedly limits glucose availability for OXPHOS, leading to impaired ATP production and excessive reactive oxygen species (ROS) accumulation (D'Alessandro et al., 2025). Accordingly, astrocytes derived from both sporadic and familial AD patient iPSCs exhibit elevated mitochondrial ROS levels and aberrant complex I activity (Bell et al., 2025).

*Impaired glycogen metabolism*: As the brain’s principal glycogen reservoir, astrocytes depend on glycogen synthase to maintain emergency energy stores. In AD, glycogen synthase activity is inhibited and glycogen levels fall (Alberini et al., 2018; Bak et al., 2018), weakening a critical metabolic buffer.

### 3.3 Collapse of metabolic output systems

The ANLS is central to metabolic coupling (Kim et al., 2025). In AD, multiple components of this metabolic output system fail. In AD, however, multiple components of this metabolic output system are disrupted. In both 3 × Tg-AD and APP/PS1 mouse models, astrocytes exhibit reduced basal lactate production derived from aerobic glycolysis, accompanied by marked downregulation of the lactate transporters MCT1, MCT2, and MCT4 relative to controls (Le Douce et al., 2020; Zhang et al., 2018). Consistently, individuals at high risk for AD show decreased MCT4 expression in the posterior cingulate cortex as early as young adulthood (Xu et al., 2016), resulting in a significant reduction in lactate availability to neurons (Wang et al., 2024). Neuronal lactate utilization is likewise compromised due to reduced LDHB expression and decreased pyruvate dehydrogenase (PDH) activity, both of which correlate with dementia severity (Jiang et al., 2023; Sakimura et al., 2024; Zhang et al., 2018).

By contrast, reactive astrocytes may, under certain conditions, upregulate glycolysis and produce excessive lactate, which can act as a signalling metabolite to amplify neuroinflammatory responses (Xiong et al., 2022). These seemingly divergent observations likely reflect differences in disease stage, experimental model or analytical methodology. Collectively, they underscore the importance of conceptualizing the ANLS as a dynamic and context-dependent pathway, rather than a static metabolic circuit, for understanding metabolic dysregulation in AD.

The glutamate–glutamine cycle, which under physiological conditions ensures efficient neurotransmitter recycling between astrocytes and neurons, is likewise driven by astrocytic glucose metabolism. Astrocytes supply both the carbon skeletons and the energetic input required for glutamine synthesis through glycolysis and the TCA cycle (Andersen and Schousboe, 2023b). In AD, this process is profoundly disrupted, resulting in impaired glutamine synthesis and pathological glutamate accumulation. The TCA cycle provides *α*-ketoglutarate as a precursor for glutamate and glutamine biosynthesis; notably, reduced TCA cycle activity and diminished glutamine production have been observed in hippocampal astrocytes from 5xFAD mice (Andersen et al., 2021b; Andersen et al., 2021c). Moreover, astrocytes exposed to amyloid-β (Aβ) display decreased expression and activity of glutamine synthetase (GS) (Andersen et al., 2022). Glutamate is the principal excitatory neurotransmitter in the central nervous system and is essential for learning and memory. In AD, Aβ enhances presynaptic glutamate release while concurrently downregulating astrocytic EAAT1/2 transporters, leading to excessive extracellular glutamate accumulation and excitotoxic neuronal injury (Andersen et al., 2022; Wu et al., 2025). Energy deficits weaken astrocytic glutamate clearance (Andersen et al., 2021b), disrupting the “glutamate uptake–lactate production” axis that normally drives ANLS coupling (Barros et al., 2023).

These failures in lactate shuttling and neurotransmitter cycling constitute a collapse of the metabolic output systems essential for neuronal survival.

### 3.4 Systemic regulatory network disruption: the role of the gut–brain axis

The gut microbiota functions as a major upstream regulator of brain metabolism, communicating with the CNS through immune, endocrine, and neural pathways (Loh et al., 2024; Wang et al., 2023). Both astrocytes and the microbiota act as highly sensitive metabolic sensors, integrating systemic metabolic cues to maintain cerebral energy balance (Zhao et al., 2021). Gut microbes modulate expression of ANLS-related genes and influence astrocytic metabolism (Margineanu et al., 2020). Probiotic supplementation restores GLUT1, GLUT3, and IGF1Rβ expression in 3 × Tg-AD mice, enhancing glucose uptake and demonstrating microbiota-driven regulation of brain glucose metabolism (Bonfili et al., 2020).

Microbial metabolites—particularly short-chain fatty acids (SCFAs)—support BBB integrity, insulin sensitivity, astrocytic glycolysis, mitochondrial respiration, and MCT1/4 expression (Jung et al., 2024; Silva et al., 2020; Zhang L. et al., 2025). SCFA supplementation rescues glutamate–glutamine cycle deficits in APP/PS1 mice (Zhang L. et al., 2025). In AD, dysbiosis alters SCFA levels and diminishes these neuroprotective effects (Silva et al., 2020). Dysbiosis-induced metabolites such as TMAO and LPS penetrate the BBB, activate toxic astrocytic programs (e.g., Smurf2/ALK5 and TLR4/NF-κB pathways), impair insulin signaling, and promote systemic inflammation (Abildinova et al., 2024; Peng et al., 2021; Haripriya et al., 2024; Su et al., 2021; Zhao et al., 2021). These changes destabilize cerebral glucose supply and further impair astrocytic metabolism. A self-reinforcing cycle emerges: disrupted gut metabolites impair astrocytes, while metabolically compromised astrocytes weaken neuronal support, promote inflammation, damage the intestinal barrier, and exacerbate microbial imbalance (Zhang L. et al., 2025). This systems-level breakdown contributes to the global collapse of the astrocytic metabolic cascade.

Astrocytic glucose metabolism in AD is disrupted at every level—from systemic gut–brain regulation to metabolic substrate input, intracellular processing, and metabolic output. This progressive breakdown shapes a pathological axis of energy deficiency that intersects with Aβ accumulation, tau pathology, synaptic failure, and neuronal degeneration.

## 4 From metabolic dysregulation to neurodegeneration: driving core pathology in AD

### 4.1 Synaptic injury and loss: the “direct executors” of cognitive decline

Synaptic dysfunction lies at the core of cognitive decline in Alzheimer’s disease (Mecca et al., 2022). Astrocytic metabolic dysfunction precipitates synaptic loss and excitotoxicity through intertwined mechanisms encompassing energy deprivation, and neurotransmitter dysregulation. In the hippocampus, reduced GLUT1 expression in hippocampal astrocytes diminishes local energy supply, leading to decreased dendritic spine density and impaired synaptic plasticity (Li Z. Y. et al., 2025), underscoring the direct impact of metabolic insufficiency on synaptic integrity.

In AD, impaired astrocytic glycolysis and the consequent reduction in lactate production compromise synaptic support. The disruption of the ANLS deprives neurons of adequate ATP. This energy deficit undermines key presynaptic processes, including the ATP-dependent proton gradient necessary for synaptic vesicle cycling and neurotransmitter loading (Faria-Pereira and Morais, 2022). Insufficient ATP also impairs Na^+^/K^+^-ATPase function, contributing to aberrant neuronal excitability (Guerra et al., 2025). Additionally, lactate acts as a signaling molecule to regulate the expression of plasticity-related genes (e.g., ARC, BDNF); its reduction may thus impair synaptic plasticity and memory consolidation (Beard et al., 2021; Suzuki et al., 2011). Astrocytic metabolic dysfunction disrupts neurotransmitter homeostasis. In AD mouse models, impaired metabolic capacity leads to reduced glutamine synthesis, thereby compromising neurotransmitter recycling and ultimately precipitating synaptic dysfunction (Andersen et al., 2021a). The synthesis of gliotransmitters such as D-serine relies on the glycolytic intermediate 3-phosphoglycerate. Downregulated L-serine levels in AD models, linked to weakened glycolysis, may impair NMDA receptor function and LTP (Orzylowski et al., 2021). Notably, elevated D-serine levels have been detected in postmortem AD brains, which may relate to dynamic changes in glycolysis during disease progression (Le Douce et al., 2020; Orzylowski et al., 2021). Astrocytes also mediate rapid clearance of excitatory neurotransmitter glutamate from the synaptic cleft via EAAT1/2 transporters, a process highly ATP-dependent (Guillem et al., 2022). ATP deficiency combined with reduced EAAT1/2 expression leads to glutamate accumulation, excessive NMDA receptor activation, calcium influx, enhanced long-term depression (LTD), and impaired LTP, collectively compromising synaptic function (Andersen et al., 2022; Popugaeva et al., 2017). Restoration of normal astrocytic glucose metabolism is therefore pivotal for maintaining synaptic integrity, supporting neuronal communication, and mitigating AD pathology.

### 4.2 Contribution of metabolic dysregulation to Aβ and tau pathology

**Figure 2:**
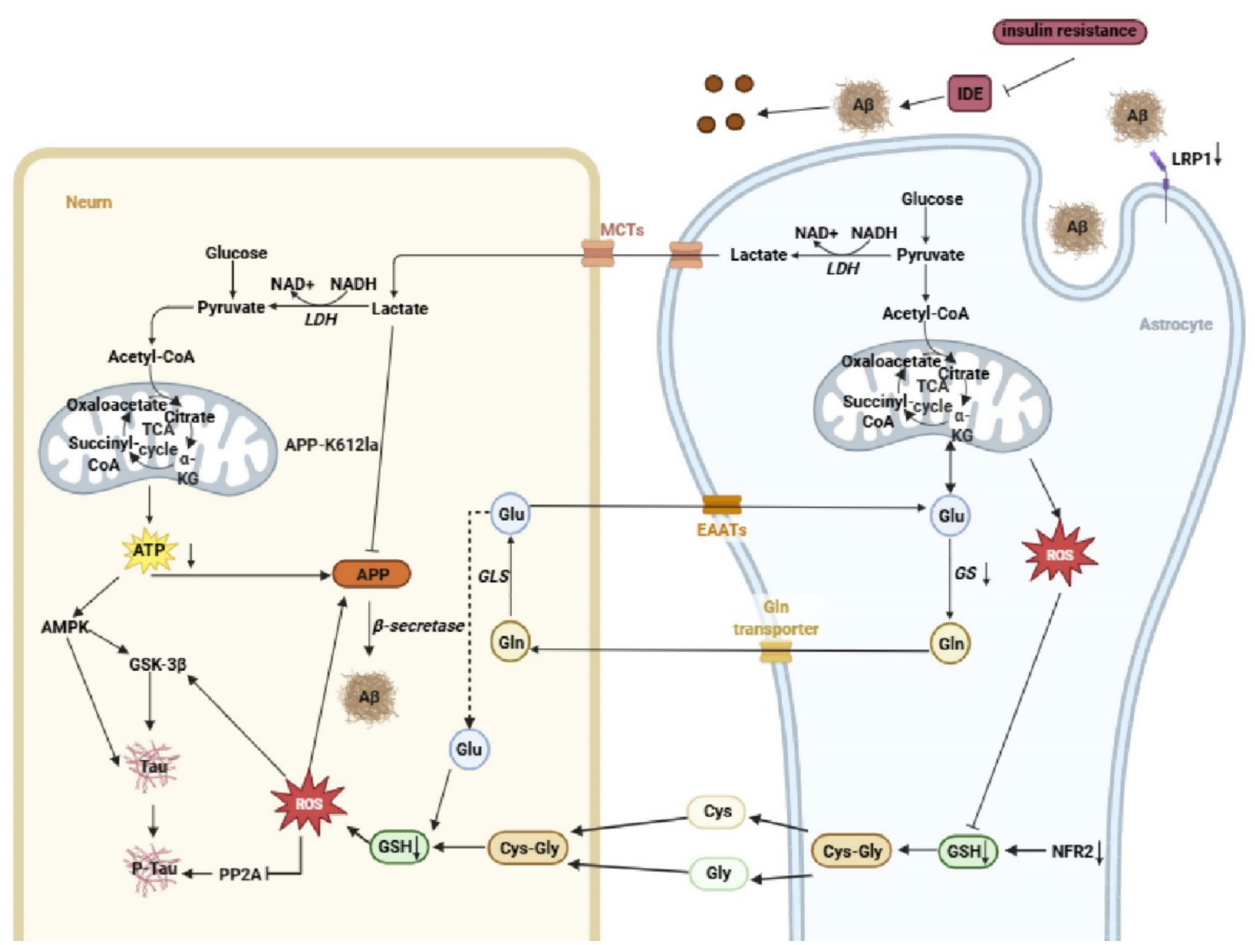
Astrocytic glucose metabolic dysregulation drives pathological Aβ and tau accumulation. Astrocytic impairment of glucose metabolism reduces lactate release, precipitating a neuronal energy deficit that promotes amyloidogenic APP processing and tau hyperphosphorylation. Insufficient lactate availability diminishes APP lactylation, thereby favoring Aβ accumulation. Concurrently, in astrocytes, elevated reactive oxygen species, suppression of the antioxidant regulator NRF2, and disruption of glutamate–glutamine cycling compromise glutathione (GSH) supply to neurons, amplifying oxidative stress and accelerating Aβ- and tau-associated pathology. Moreover, insulin resistance–mediated downregulation of insulin-degrading enzyme (IDE) and low-density lipoprotein receptor–related protein 1 (LRP1) further impairs Aβ clearance, reinforcing pathogenic protein accumulation.

In AD, Aβ/tau pathology and metabolic dysfunction mutually reinforce each other. Studies show that inhibiting the key glycolytic regulator PFKFB3 in astrocytes (e.g., with 3PO/PFK15) increases Aβ accumulation and plaque formation (Fu et al., 2015); whereas inhibiting the astrocytic pyruvate carrier in 3xTgAD mice reduces amyloid and tau deposition (Ceyzeriat et al., 2024). These findings suggest that astrocytic glucose metabolism can drive AD pathological progression, likely through indirect mechanisms involving the exacerbation of neuronal energy crisis and oxidative stress (Figure 2).

Impaired astrocytic glycolysis and the consequent disruption of the ANLS lead to neuronal ATP deficiency and an energy crisis. This energetic state directly influences amyloid precursor protein (APP) processing: under sufficient energy, APP is primarily cleaved via the non-amyloidogenic pathway (*α*/*γ*-secretase) into soluble fragments; whereas under energy deficit, it tends to be processed through the amyloidogenic pathway (β/γ-secretase), generating full-length Aβ peptides (Blonz, 2017). The energy crisis also activates AMPK. While AMPK activation may help reduce Aβ production, it can also directly or indirectly (via GSK-3β activation) lead to tau hyperphosphorylation, promoting neurofibrillary tangle formation (Marinangeli et al., 2016; Khandelwal et al., 2022; Yu et al., 2025). Glycolytic products such as lactate exert bioactivity via post-translational modifications; for instance, lactylation of APP at K612 attenuates its interaction with BACE1, reducing Aβ generation. In AD patients and mouse models, APP-Kla levels are markedly decreased, and exogenous lactate supplementation can suppress Aβ production (Tian et al., 2025), an effect attenuated in advanced disease stages due to diminished glycolysis and lactate availability. Astrocytic antioxidant capacity is also compromised in AD, with reductions in GSH levels. Neuronal GSH synthesis relies on cysteine and glycine derived from astrocytic GSH degradation, as well as glutamine provision by astrocytes (Dringen and Arend, 2025). In AD, downregulation of the key transcription factor Nrf2 further impairs astrocytic GSH production, while glucose metabolic deficits reduce glutamine availability (Andersen et al., 2022; Chu et al., 2024; Osama et al., 2020). Accumulating ROS further deplete astrocytic GSH reserves, impairing neuronal antioxidant defenses and contributing to oxidative stress intimately linked to AD pathology (D'Alessandro et al., 2025). ROS elevation enhances β- and γ-secretase cleavage of APP, favoring aggregation-prone Aβ species (D'Alessandro et al., 2025; Huang et al., 2020). Oxidative stress often precedes Aβ and tau pathology, and mitochondrial ROS promotes tau oligomerization and accumulation (Du et al., 2022; Roy et al., 2023). Excess ROS inhibit protein phosphatase 2A (PP2A), a primary astrocytic tau phosphatase, while activating GSK-3β, collectively driving tau hyperphosphorylation and NFT formation (D'Alessandro et al., 2025). The energy crisis and metabolic dysregulation also impair Aβ clearance mechanisms. Insufficient ATP supply may disrupt the autophagy-lysosomal pathway (Falace et al., 2024). In 5xFAD mice, loss of insulin signaling in astrocytes enhances tau phosphorylation and impairs Aβ clearance (Chen W. et al., 2023). Hyperglycemia and insulin resistance resulting from impaired insulin signaling competitively inhibit Aβ degradation by insulin-degrading enzyme (IDE) and downregulate the expression of the key astrocytic Aβ clearance receptor LRP1, collectively reducing Aβ clearance (Faissner, 2023; Sedzikowska and Szablewski, 2021; Li W. et al., 2025; Shinohara et al., 2017).

Notably, PET imaging indicates that in late-stage AD, glucose hypometabolism correlates with tau burden but not Aβ load (van Gils et al., 2024). While numerous studies link regions of metabolic impairment with Aβ and tau deposition, mechanistic delineation of how astrocytic metabolic dysfunction directly drives these pathologies remains limited and warrants further investigation.

### 4.3 Convergence points and the vicious cycle

In AD, astrocytes undergo reactive transformation, exhibiting high phenotypic and functional heterogeneity that extends beyond the simplistic A1 (neurotoxic)/A2 (neuroprotective) binary classification (Xiong et al., 2022). The A1 phenotype can directly damage neurons by releasing complement components (e.g., C3), pro-inflammatory factors, and reactive oxygen species, while losing their normal synaptic support functions (Liddelow et al., 2017; Xiong et al., 2022). Furthermore, reactive astrocytes may also influence neuronal network excitation-inhibition balance through GABA secretion (Garaschuk and Verkhratsky, 2019). Conversely, the A2 phenotype is capable of releasing neurotrophic factors and aiding in the clearance of pathological protein aggregates (Liddelow and Barres, 2017).

Current evidence indicates that augmented glycolysis in astrocytes is a pivotal driver of their reactive activation in AD, fostering a self-reinforcing pro-inflammatory feed-forward loop (Bell and Mortiboys, 2026; Sanchez et al., 2025). Notably, dual PET tracer imaging has demonstrated a close association between astrocytic activation and heightened glycolytic activity (Carter et al., 2019). Evidence indicates that Aβ-activated microglia indirectly induce A1 astrocyte formation, a process dependent on upregulated astrocytic glycolysis (Zhang et al., 2022). Glycolytic flux is critical for activating neurotoxic astrocytes, and its inhibition in APP/PS1 models suppresses A1 reactivity (Yang X. et al., 2024). The glycolytic regulator PFKFB3 sustains reactive astrocyte metabolism and function (Calì et al., 2024). Notably, increased glycolysis in AD can paradoxically fuel inflammation by promoting astrocytic activation, forming a pathogenic cycle. Studies in primary astrocytes confirm that NF-κB-driven inflammatory responses are glycolysis-dependent (Robb et al., 2020). PFKFB3 overexpression in reactive astrocytes drives a Warburg-like shift, leading to lactate accumulation that exacerbates neuroinflammation (Fang et al., 2024; Xiong et al., 2022). Excess lactate may also contribute to tau pathology, for instance by promoting lactylation at the K331 site, thereby exacerbating tau misfolding and aggregation. Inhibition of LDHA expression has been shown to ameliorate this process (Zhang X. et al., 2025). Furthermore, mitochondrial dysfunction participates in modulating the pro-inflammatory phenotype of astrocytes. The mitochondrial fission protein DRP1 promotes inflammatory factor release by driving mitochondrial fission and activating the NF-κB pathway (Cai et al., 2024; Sbai et al., 2023). Inhibition of DRP1-mediated mitochondrial fission has been proven to mitigate astrocyte-derived inflammation (Wu et al., 2020).

Microglial metabolic reprogramming further interweaves with astrocytic dysfunction (Sangineto et al., 2023). M1 microglia secrete IL-1*α*, TNF-α, and C1q, inducing resting astrocytes into neurotoxic A1 states (Singh, 2022), or directly stimulate A1 astrocytes via IL-1*β*, TNF-α, and IL-6 (Wu and Eisel, 2023). Reciprocally, activated astrocytes modulate microglial states, secreting IL-1β, CCL2, and CXCL10 to promote M1 microglial activation (Kwon and Koh, 2020; Wu and Eisel, 2023). This glial crosstalk fosters neuroinflammation, which in turn serves as a potent stimulus for glial activation, establishing a self-perpetuating vicious cycle. Pro-inflammatory stimuli (e.g., LPS, IL-1β/TNF-α) enhance glial activation and astrocytic glycolysis, and can induce lasting epigenetic changes in astrocytes (Linnerbauer et al., 2020; Pamies et al., 2021; Xingi et al., 2023). Together, this metabolic-inflammatory vicious cycle drives AD progression.

## 5 Therapeutic potential of targeting the glycolytic cascade

Targeting the astrocytic glycolytic cascade represents a promising therapeutic strategy for AD (Table 2). Lifestyle and dietary modifications show beneficial effects: a ketogenic diet can alleviate AD symptoms, potentially by restoring TCA cycle activity and promoting aerobic glycolysis; however, whether these effects are astrocyte-specific remains unclear (Hersant and Grossberg, 2022; Yu et al., 2022). Intermittent fasting enhances hippocampal insulin signaling and suppresses Aβ deposition (Park et al., 2020); moderate-to-high-intensity exercise augments astrocytic aerobic glycolysis, modulates ANLS-related protein expression, and improves mitochondrial function (Xue et al., 2022). Notably, existing studies on lifestyle and dietary interventions are generally limited in scale, and robust, long-term randomized controlled trials assessing their efficacy in AD patients are still lacking.

**Table 2:** Interventions targeting astrocyte glucose metabolism cascades and their effects in AD.

Intervention category	Intervention	Effect	Refs.
Lifestyle	Ketogenic diet	Ameliorates AD progression; restores TCA activity; ↑acetyl-CoA; promotes aerobic glycolysis in brain	Hersant and Grossberg (2022), Yu et al. (2022)
	Intermittent fasting	Improves memory; enhances hippocampal insulin signaling; ↓Aβ deposition	Park et al. (2020)
	Moderate-to-high intensity exercise	↑Astrocytic aerobic glycolysis; regulates ANLS transporters and enzymes; slows AD-like pathology	Xue et al. (2022)
Pharmacological/biological	Intracerebral insulin + oral cinnamon extract	Improves cognition; ↑GLUT1 expression	Sajadi et al. (2023)
	GLP-1R agonists (e.g., Tirzepatide)	Rescues cognitive deficits; prevents neurodegeneration; ↑GLUT1, HK, G6PD, PFK mRNA	Yang S. et al. (2024)
	Lonsamin, Phenformin, Berberine	↑Astrocytic glucose uptake and lactate production; enhances glycolysis; ↑ApoE lipidation; ↓Aβ plaques	Patil and Kuehn (2024), Sun et al. (2023)
	Drp1 inhibitors (Mdivi1, P110, DDQ)	Corrects mitochondrial dynamics; improves energy metabolism	Bhatti et al. (2023)
	Antioxidant (SKQ1)	Limits ROS-induced neuronal damage; anti-inflammatory	Rudnitskaya et al. (2022)
	IDO1 inhibitor	↑Glucose metabolism and spatial memory; restores lactate transfer	Minhas et al. (2024)
	PDHK inhibitor	↑Pyruvate and glucose utilization; ↑lactate production; activates ANLS; neuroprotection	Sakimura et al. (2024)
	Sulbactam	↑EAAT2; enhances neuronal tolerance to Aβ and glutamate toxicity	Li et al. (2024)
	MnO₂ nanoparticles	↑Glucose utilization; improves glucose metabolism and mitochondrial activity; enhances long-term neuronal synaptic function	Park et al. (2025)
Gut–brain axis	Microbiota transplantation + prebiotics	↑Atp1a2/Pfkfb3 expression	Margineanu et al. (2020)
	Rheum anthraquinones, β-sitosterol	Inhibits TMA production by blocking TMA-lyase; ↓TMA	Wu et al. (2022)

Pharmacological interventions focusing on key steps of glucose metabolism have been extensively investigated. Intracerebral insulin delivery and oral administration of cinnamon extract have been reported to improve cognitive performance and increase GLUT1 protein expression in AD rat models, although astrocyte-specific effects have not been conclusively demonstrated (Sajadi et al., 2023). GLP-1 receptor agonists and the dual GLP-1/GIP receptor agonist Tirzepatide increased cortical mRNA expression of GLUT1, HK, G6PD, and PFK in APP/PS1 mice (Yang S. et al., 2024). Compounds such as *Lonicera nitida*, phenformin, and berberine stimulate glucose uptake and lactate production in astrocytes, enhance ApoE lipidation, and may thereby accelerate Aβ clearance (Patil and Kuehn, 2024; Sun et al., 2023). To address abnormal mitochondrial dynamics, Drp1 inhibitors (e.g., Mdivi1, P110) improve energy metabolism (Bhatti et al., 2023; Zysk et al., 2023), while the mitochondria-targeted antioxidant SkQ1 mitigates ROS-related neuronal damage (Rudnitskaya et al., 2022). Additionally, IDO1 inhibitors improve hippocampal glucose metabolism and lactate transfer (Minhas et al., 2024); a novel PDHK inhibitor enhances lactate production in astrocytes and activates ANLS (Sakimura et al., 2024); and suberoylanilide hydroxamic acid upregulates EAAT2, increasing neuronal tolerance to toxicity (Li et al., 2024). Multifunctional manganese dioxide nanoparticles have been shown to enhance brain glucose utilization and mitochondrial activity (Park et al., 2025).

Gut microbiota regulation offers another potential avenue. Fecal microbiota transplantation and prebiotic supplementation upregulate ANLS-related gene expression (Margineanu et al., 2020). Substances that inhibit TMA production, such as rhubarb anthraquinones and β-sitosterol, may ameliorate astrocytic energy metabolism dysfunction via the gut-brain axis (Wu et al., 2022). In summary, multi-level interventions—through lifestyle, pharmacology, and microbiota modulation—aimed at regulating the astrocytic glycolytic cascade provide a novel framework for AD treatment, though further research is required for clinical translation.

## 6 Conclusion and perspectives

This review systematically delineates the dysregulation of the astrocytic glycolytic cascade in AD, characterized by a comprehensive disruption spanning “input–pathway–output.” This derangement impairs astrocytic neuroprotective functions, damages synapses, exacerbates Aβ/tau pathology, and drives the conversion of reactive astrocytes toward neurotoxic phenotypes, thereby establishing a “metabolic dysfunction–neuroinflammation” vicious cycle central to AD progression. Furthermore, gut microbiota and their metabolites remotely modulate astrocytic metabolism via the gut–brain axis, revealing an interplay between systemic and central metabolic disturbances. Based on this, we propose a “central astrocytic metabolic collapse” model. This model situates astrocytic metabolism within a complex network involving the gut–brain axis, cerebrovasculature, neurons, and microglia, positing AD as a multifaceted disease driven by metabolic collapse and sustained by multiple positive-feedback loops.

Notably, astrocytes serve not only as key regulators of cerebral energy metabolism but also as central hubs of lipid metabolism. They synthesize cholesterol, load it onto APOE to form lipoprotein particles, and then transport these particles to neurons to maintain their normal function (Liu et al., 2025). Astrocytes are the primary producers of APOE in the brain; the APOE4 isoform binds with high affinity to triglyceride-rich very-low-density lipoproteins (VLDL), leading to downregulation of low-density lipoprotein receptors (LDLRs) and disruption of lipid homeostasis. Perturbations in lipid metabolism, in turn, further constrain astrocytic glucose metabolic capacity, reducing overall energetic efficiency. Mechanistically, APOE4 diminishes insulin–insulin receptor interactions, suppressing activation of the downstream Akt/GSK3β signaling axis and thereby impairing intracellular glucose metabolism (Budny et al., 2025b; Zhao et al., 2017). In humanized APOE4 mouse models, APOE-driven global metabolic reprogramming has been characterized by a shift away from glucose utilization toward lipid oxidation (Arbones-Mainar et al., 2016). Complementary studies in hiPSC-derived astrocytes have demonstrated that APOE4 directly perturbs key glycolytic enzymes while simultaneously impairing oxidative phosphorylation through lysosomal cholesterol accumulation, increased mitochondrial proton leak, and enhanced mitochondrial fusion. These alterations elicit compensatory glycolytic activation and are accompanied by pro-inflammatory and senescent phenotypes (Budny et al., 2025a; Caceres-Palomo et al., 2025; Lee et al., 2023). Moreover, hiPSC-based analyses reveal that APOE4 compromises astrocytic Aβ phagocytosis, thereby accelerating disease progression (Lee S. et al., 2025). Collectively, these findings underscore that astrocytic metabolic collapse in AD represents a failure of integrated metabolism, in which glucose and lipid metabolic dysfunctions converge through APOE4 as a critical amplifier. Accordingly, future investigations into astrocytic lipid metabolism are as essential as those focused on glucose metabolism.

Current data have systematically revealed extensive remodeling of astrocytic glucose metabolism in the brains of AD patients and related models, encompassing widespread disruption of the glycolytic cascade. In parallel, multiple studies have demonstrated that astrocytes differentiated from induced pluripotent stem cells derived from patients with late-onset AD harbor cell-intrinsic metabolic abnormalities, including attenuated INS/IGF-1 signaling, inefficient glycolytic activation, and reduced NAD^+^ availability (Cohen and Sonntag, 2023; Ryu et al., 2021a; Ryu et al., 2021b). Collectively, these observations imply that the pathophysiological substrate of a subset of AD cases may be established as early as neurodevelopment. Nevertheless, metabolic phenotypes reported across different model systems remain highly variable and, at times, contradictory. In particular, whether astrocytic glycolysis is ultimately upregulated or suppressed in AD remains unresolved, a discrepancy that likely reflects both intrinsic model-dependent differences and the pronounced heterogeneity of disease stage. Therefore, extrapolating findings from individual models to the human AD trajectory requires considerable caution. Future efforts should prioritize the establishment of cross-model validation pipelines and the use of iPSC-based systems and brain organoids to more faithfully recapitulate human genetic backgrounds and disease timelines. Although astrocytic glucose metabolic disturbances are spatiotemporally associated with core AD pathologies, establishing unequivocal causality remains a central challenge. Much of the existing evidence is correlative, such as the spatial overlap between metabolic abnormalities and Aβ deposition or transcriptomic alterations in metabolic pathways. These observations imply association but cannot distinguish whether metabolic dysfunction is a cause, consequence, or parallel feature of AD pathology. By contrast, interventional studies offer more causal insight. For example, pharmacological inhibition of the astrocytic glycolytic regulator PFKFB3 exacerbates Aβ pathology in animal models, whereas enhancing glycolysis or lactate output can, under specific conditions, improve neuronal survival and cognitive performance. It is noteworthy that lactate accumulation resulting from aberrantly enhanced glycolysis may itself influence AD progression by promoting protein lactylation, an emerging epigenetic regulatory mechanism. Through this pathway, metabolic disturbances can be transduced into sustained alterations in gene expression and cellular function, positioning lactylation as a potential signaling bridge linking astrocytic metabolic dysregulation to functional impairment in AD (Gu et al., 2026). Nevertheless, most such interventions lack cell-type specificity and temporal precision. To date, few studies have achieved reversible, astrocyte-specific manipulation of discrete metabolic pathways at defined stages of AD progression while systematically assessing downstream effects on Aβ/tau accumulation, synaptic plasticity, and behavioral outcomes.

Despite the initial recognition of the central role of the astrocytic glucose metabolic cascade in AD, current research still faces several key bottlenecks. Astrocytes are a highly heterogeneous cell population, and their metabolic phenotypes exhibit distinct regional specificity and disease-stage dependence. Glycolytic processes undergo dynamic changes during disease progression, and the specific mechanisms underlying these changes remain unclear. Different intervention strategies may be required during phases of metabolic hyperactivation versus metabolic exhaustion (Preman et al., 2021; Wei et al., 2024). Future research should establish human iPSC-derived cells and organoids as core validation platforms to confirm mechanisms identified in animal models and to prioritize human-specific therapeutic targets. Animal models, in turn, should be leveraged primarily to test the efficacy of interventions at the level of integrated systems. Given the complexity and redundancy of metabolic networks, targeting single molecules such as GLUT1 or PFKFB3 may yield limited efficacy or incur off-target effects. Instead, next-generation therapeutic strategies should explore combinatorial approaches, such as concurrently restoring astrocytic glucose metabolism while alleviating lipotoxicity or inflammatory signaling (for example, by modulating APOE lipidation or inhibiting specific inflammatory pathways), or integrating metabolic modulators with conventional Aβ- or tau-directed therapies.

In summary, astrocytic metabolic dysfunction constitutes a critical and indispensable hub within the AD pathological network. Although substantial evidence supports its central role, our understanding of its precise mechanistic contributions, causal hierarchy, and spatiotemporal dynamics remains incomplete. By deploying more refined experimental tools, integrating evidence across multiple model systems, and rigorously dissecting causal chains, targeting the astrocytic glycolytic cascade holds promise for breaking the current impasse in AD therapeutic development and for enabling a paradigm shift from symptomatic relief toward true disease modification.
